# Fabrication of Random Microwell Arrays as Pseudo-Thermal Speckle Light Source

**DOI:** 10.3390/mi9060256

**Published:** 2018-05-24

**Authors:** Axiu Cao, Hui Pang, Jiazhou Wang, Lifang Shi, Qiling Deng, Song Hu

**Affiliations:** 1 Institute of Optics and Electronics, Chinese Academy of Sciences, Chengdu 610209, China; longazure@163.com (A.C.); ph@ioe.ac.cn (H.P.); wuli041@126.com (J.W.); dengqiling@ioe.ac.cn (Q.D.); husong@ioe.ac.cn (S.H.); 2 University of Chinese Academy of Sciences, Beijing 100049, China

**Keywords:** microfabrication, microwell arrays, speckle light source, pseudo-thermal source

## Abstract

Quantum correlated imaging using the intensity fluctuations of thermal light possesses advantages of high resolution and strong anti-interference ability. The common method to produce pseudo-thermal light source is using a rotary ground glass and transmission of laser beam. In the present work, we propose a method for the fabrication of microwell arrays with randomly varied diameters, which could be used as a new structural element for pseudo-thermal speckle light source. If these are etched with random sizes then they may also have random and complex varying curvatures (diffusion limited etching) leading to random destructive interference of the coherent beam which could be a good thing. The microwell arrays, with diameters randomly varying from 5 μm to 40 μm, height varying from 200 nm to 20 μm, were fabricated by photolithography combined with acid etching. The experimental conditions are simple and can be scaled up to for large structures. The produced microwell arrays can transform the laser beam to a pseudo-thermal light source with a certain divergent angle by rational designing of mask and adjustable process parameters.

## 1. Introduction

Quantum correlated imaging is a new type of imaging using quantum entanglement or intensity fluctuation characteristics of light field [1,2,3,4]. Owing to its high resolution and strong anti-interference ability, thereby has potential applications in life science, information technology and national defense equipment development.

The pioneer researchers produced entangled photon pairs to carry out ghost imaging [5,6,7] and regarded entangled light sources as the basis of realization of ghost imaging. However, further experiments suggested that pseudo-thermal light could also be used as a light source to achieve ghost imaging with higher resolution [8,9,10,11]. There are two characteristics of the thermal light field: (1) the intense random fluctuations of the thermal field; (2) the complex amplitude of the thermal field obeys the Gauss statistics. The thermal light source, which satisfies the thermal optical correlation of Gauss statistics, can imitate the entangled light source for correlation imaging. The thermal light source is easily obtained, but its coherence time is usually short or the intensity of the light is ordinarily weak. So, the detectivity is reduced by the ordinary detector. In practical application, the laser light beam can be modulated by different methods to imitate the thermal light field. The pseudo-thermal light source can be obtained in the far field through coherent superposition, which results in a beam that exhibits fluctuation in space and time. Therefore, the pseudo-thermal light is more readily obtained, which reduces the difficulty and cost of ghost imaging.

State-of-the art of pseudo-thermal light source is typically produced by the laser transmitting through a rotary ground glass [12,13,14]. When quantum correlated imaging technology is applied in remote sensing, missile-borne, or satellite-borne remote imaging, higher requirements for the pseudo-thermal light source are needed. These includes small divergence angle of the whole light, no center zero-order point, energy be effectively concentrated in a certain region in long-distance transmission. The laser speckle field modulated by ground glass is through the irregular distribution of fine particles, so its divergence angle is always dozens of degrees and uncontrollable.

In this work, we propose the fabrication of microwell arrays with randomly varied diameters as a new structure as pseudo-thermal speckle light source. The microwell array can be fabricated by photolithography combined with acid etching, and the obtained pseudo-thermal source can be generated with a controllable divergent angle.

## 2. Experimental Section

The structure microwell array was composed of randomly distributed numerous microwells, which serve as independent scattering centers. The light fields scattered by the random independent scattering centers were superimposed on each other to form the final speckle field.

### 2.1. Fabrication Method

**Figure 1:**
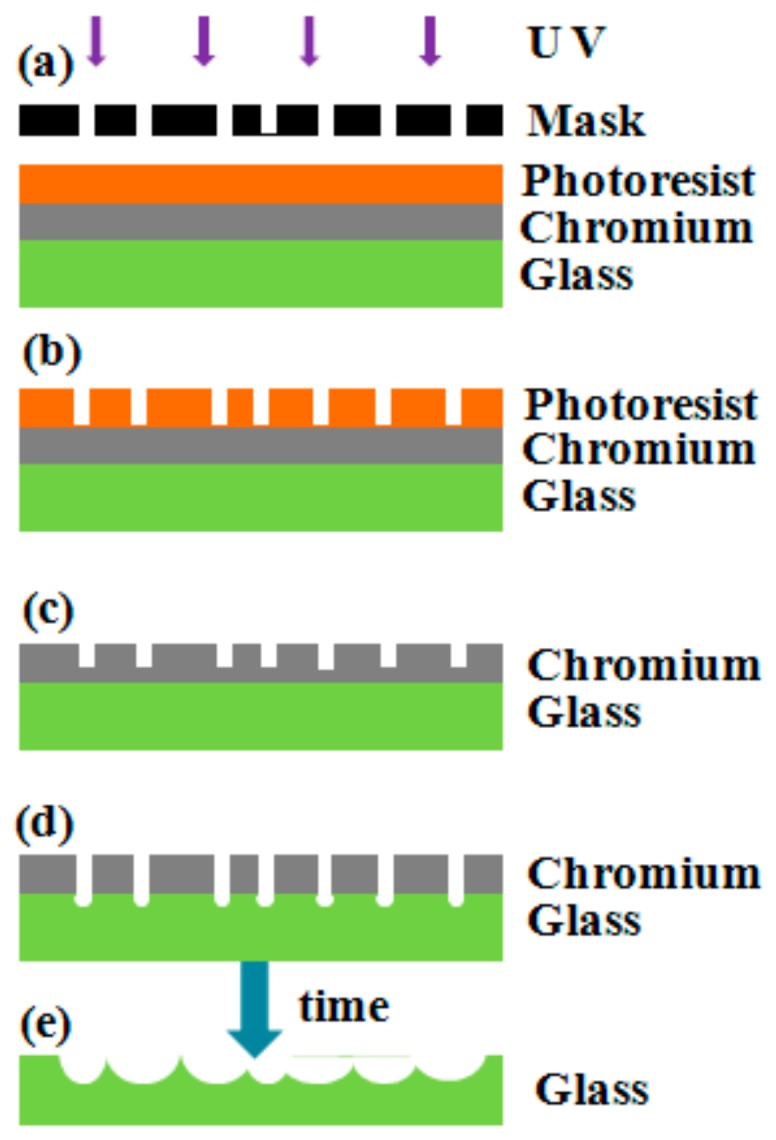
The procedure of fabricating randomly distributed microwell arrays: (**a**) a glass with chromium plating was used as substrate, and exposure was carried out with designed mask; (**b**) photoresist-based structure was achieved after development; (**c**) the photoresist pattern was transferred to the surface of the chromium film; (**d**) acid etching; (**e**) random distribution of microwell array.

Randomly distributed microwell array was fabricated by photolithography combined with acid etching (Figure 1). Firstly, a glass with chromium plating was selected as the substrate. The photoresist was spun on the surface of the glass as the initial structure. Secondly, a mask with randomly distributed patterns composed of polygon was designed for lithography exposure technology (see Figure 1a). The patterns were then transferred to the photoresist layer by exposure, development, and post baking (see Figure 1b). Thirdly, the photoresist pattern was transferred to the surface of the chromium film through the etching with solution. By controlling the time of chromium removal etching, the corrosion of chromium film with a certain thickness was processed until the chromium layer changed into a pseudo-random state (see Figure 1c). Finally, the photoresist was removed. The substrate was put into the hydrofluoric acid solution for etching, and a random distributed microwell array was obtained (see Figure 1d,e).

### 2.2. Fabrication Process

**Figure 2:**
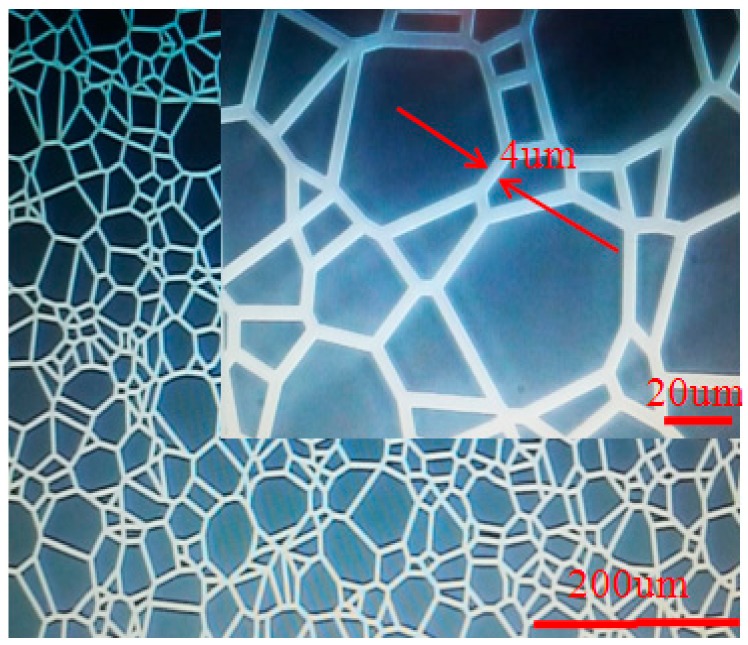
An enlarged photograph showing the pattern of a prepared mask.

The designed mask was composed of randomly distributed polygon structure. The polygon area was opaque with size varying from 5 μm to 40 μm. The gap between the polygons was 4 μm. The enlarged view of the as prepared mask is shown in Figure 2.

A glass was selected as the substrate followed by a series of surface pretreatment. First, the substrate was immersed in acetone for 10 min to remove organic matter on its surface. Then, it was taken out to soak in alcohol for 10 min to remove the remained acetone. Subsequently, the surface of the substrate was washed with deionized water and dried with nitrogen. The cleaned substrate was physically baked for 20 min on a hot plate or in an oven to evaporate water.

**Figure 3:**
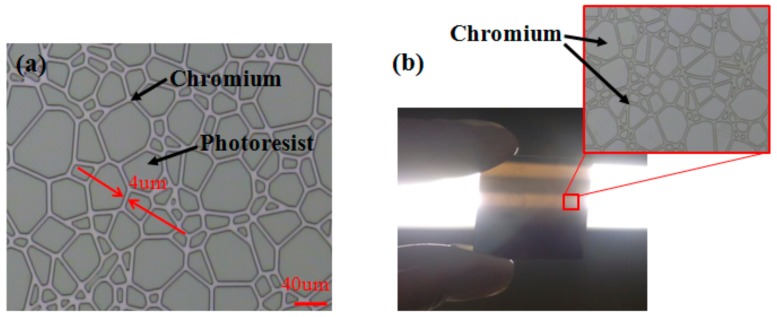
Mask pattern was transfer to (**a**) photoresist layer (**b**) chrome layer.

Surface of the substrate was vapor-deposited with a chromium layer about 100 nm thick as a mask for subsequent patterns. A layer of photoresist (AZ MIR 703 Photoresist (14cP), Merck Electronic Materials, Shanghai, China) was then spin-coated on chromium layer. The spin-coated time was 20 s at a speed of 3000 rpm. Key parameters of prebake temperature, prebake period, and the obtained photoresist thickness were 90 °C, 20 min, and 1.1 μm, respectively. The UV lithography machine (URE-2000S/A) with wavelength of 365 nm was used. The exposure power density of exposure machine was adjusted to 3.2 mw/cm^2^. The photoresist was exposed for 20 s using the designed mask by the contact exposure method. Then, the exposed substrate was developed in a developing solution (AZ300 MIF Developer, Merck Electronic Materials, Shanghai, China) for 30 s to obtain a resist pattern structure, as shown in Figure 3a. Here, the chromium layer was exposed in the gaps between polygons.

The substrate with exposed chromium layer was immersed in solution (solution/deionized water = 1:2) for 45 s. The non-photoresist-masked chromium layer was attenuated by solution till the substrate showing a pseudo-random state, as shown in Figure 3b. The amplified chromium layer pattern is shown in the box.

**Figure 4:**
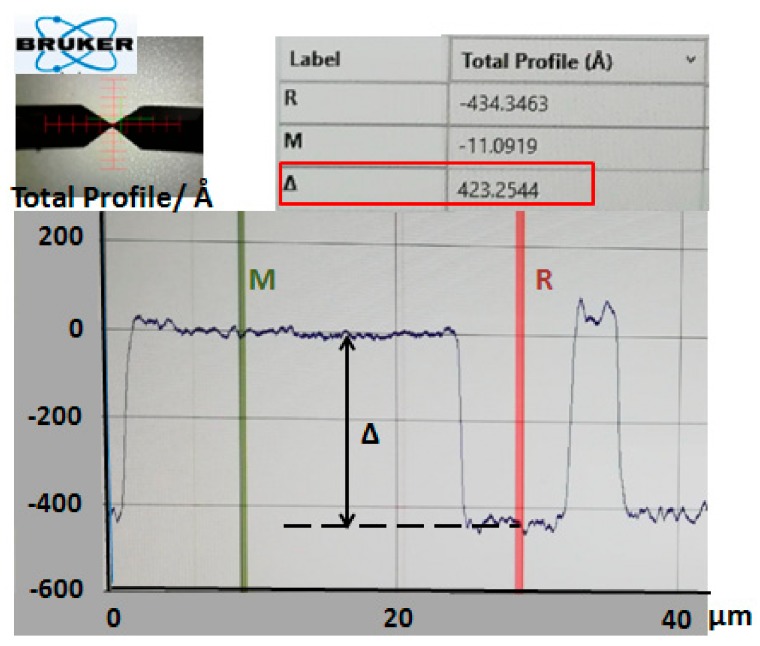
The surface profile of chrome layer.

The depth Δ of the chrome layer pattern was 42.3 nm, measured by a step analyzer (Stylus Profiler System, Dektak XT, Bruker, Karlsruhe, Germany) (Figure 4).

Finally, the substrate was immersed in an acidic etching solution (hydrofluoric acid/nitric acid/glacial acetic acid/deionized water = 1:1:3:10) to perform glass etching to produce randomly distributed microwell arrays.

## 3. Preparation Results and Experiment

**Figure 5:**
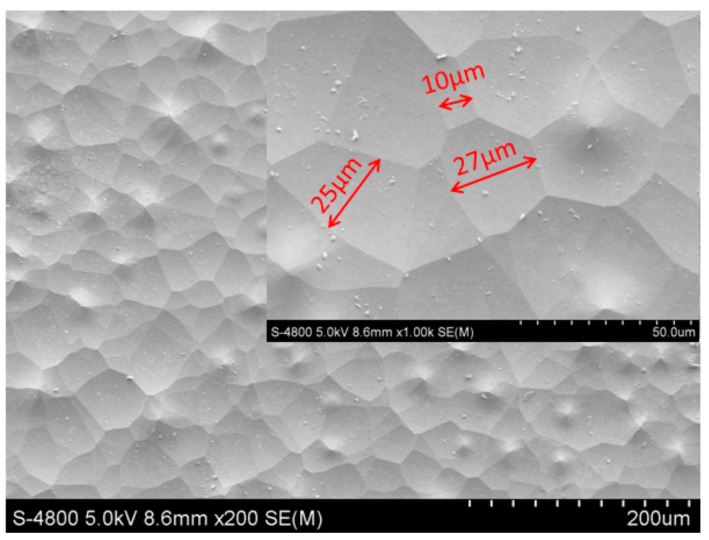
Micrograph of the microwell array.

**Figure 6:**
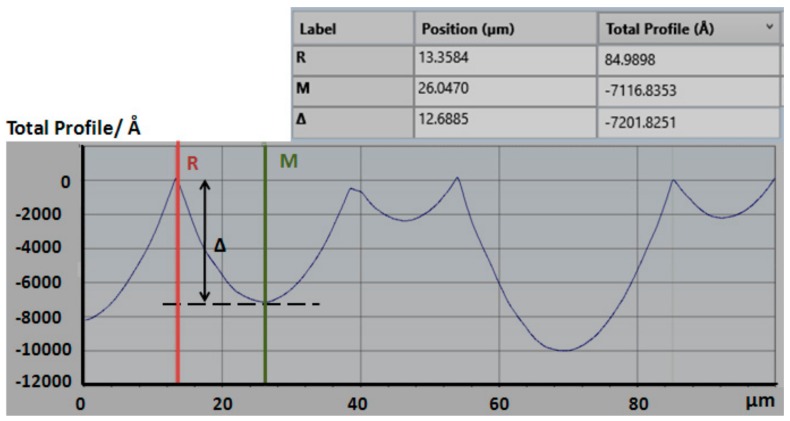
The surface profile of the microwell array.

The prepared microwell array with size of 60 mm × 60 mm × 1 mm was measured by scanning electron microscope (SEM, Hitachi S-4800, Hitachi, Ltd., Tokyo, Japan). As shown in Figure 5. The surface of the substrate is arranged of different microwell arrays with sizes within the range of tens of micrometers. The depth of the microwell was 720 nm, measured by a step analyzer (Figure 6). By repetitious measurement, the height is statistically varied in range of 200 nm to 20 μm. Based on the least square method, the profile data was fitted to calculate the root mean square roughness, which is 2.4 nm.

**Figure 7:**
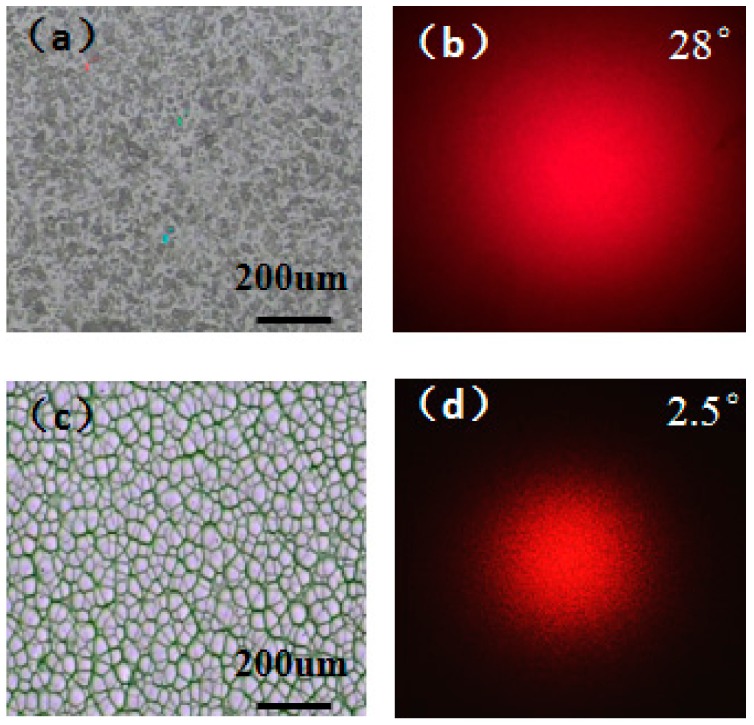
The surface profiles of ground glass and prepared microwells along with the speckle patterns for comparison: (**a**) the surface profile of ground glass; (**b**) the speckle pattern produced by ground glass; (**c**) the surface profile of prepared microwells; (**d**) the speckle pattern produced by prepared microwells.

The optical test experiment was carried out to validate the performance of the as prepared structure. The traditional random ground glass and the prepared microwells were vertically irradiated by a laser with a wavelength of 650 nm, and the random speckle patterns were tested at the distance of 29.5 cm, respectively. The surface profiles measured with a microscope (BX51, OLYMPUS, Shanghai, China) of ground glass (Figure 7a) and prepared microwells (Figure 7c) along with the speckle patterns were achieved. The diameter of the speckle caused by ground glass was about 14.7 cm and the divergence angle of the light field was calculated to be 28° (Figure 7b). The diameter of the speckle produced by prepared microwells was about 1.28 cm. The divergence angle of the light field was calculated to be 2.5° (Figure 7d). The laser speckle field modulated by ground glass is through the irregular distribution of fine particles. However, the proposed component can be seen as a random distribution of multiple micro-lenses with concave surface; that means, for given single microwell, the surface of the well will result in an associated deflection. The deflection should be power independent but the associated randomization of the phase from the microwell will change the resultant beam deflection. All light beams are suppression to generate the pseudo-thermal speckle light source by averaged the deflected light beam. Due to the laser speckle field modulated by ground glass through the irregular distribution of fine particles within the size of 10 nm, its divergence angle is bigger than the structure proposed here. 

**Figure 8:**
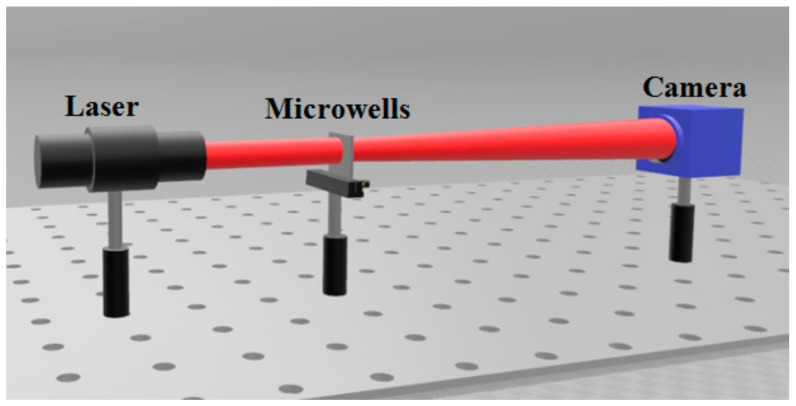
Layout of the spackle measurement.

**Figure 9:**
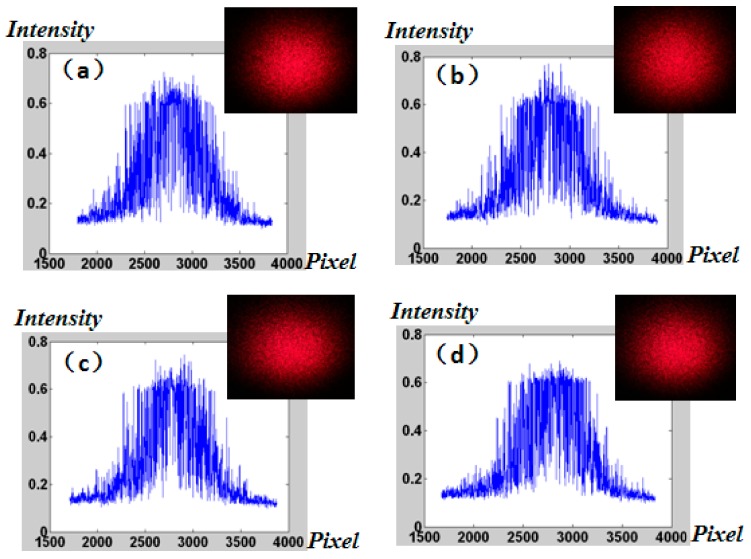
The random speckle patterns at different locations produced by prepared microwells: (**a**) up; (**b**) down; (**c**) left; (**d**) right.

The random speckle patterns at different locations produced by prepared microwells were achieved in detail by using the HR16000CTLGEC camera with resolution of 4896 × 3248 and pixel size of 7.4 μm × 7.4 μm (Figure 8). The patterns are shown in Figure 9. Comparing the energy distribution of speckle at four different locations, it was found that the number of pixels in the speckle pattern diameter is approximately equal to 1730. According to the size of the pixel of 7.4 μm, the diameter of the pattern is 12,802 μm. So, the divergence angle of the speckle is further verified to be 2.5°.

**Figure 10:**
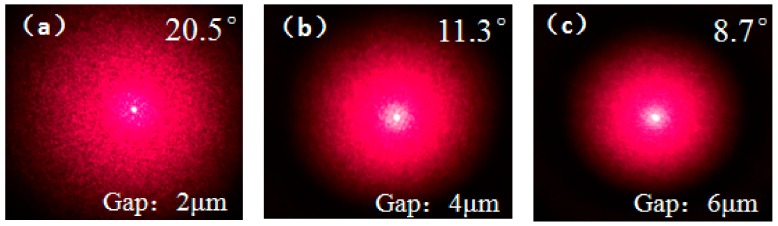
The scattering speckle patterns produced by the mask with different sizes of gaps: (**a**) 2 μm; (**b**) 4 μm; (**c**) 6 μm.

By changing the mask parameters, the influence on the speckle divergence angle of the structure was analyzed. The gaps between the polygons were designed as 2 μm with a diameter range of 2.5 μm to 20 μm, 4 μm with a diameter range of 5 μm to 40 μm, and 6 μm with a diameter range of 7.5 μm to 60 μm, respectively. The size distributions of the polygon patches were designed according to the proportion of size of the gaps. The scattering speckle patterns produced by the mask were tested as 20.5°, 11.3°, and 8.7°, respectively, as shown in Figure 10. It was found that the size of speckle and the divergence angle decreases along with the increscent size of the gaps and polygon patches. It can be seen that the feature size of polygon affects the final divergence angle of the speckle.

**Figure 11:**
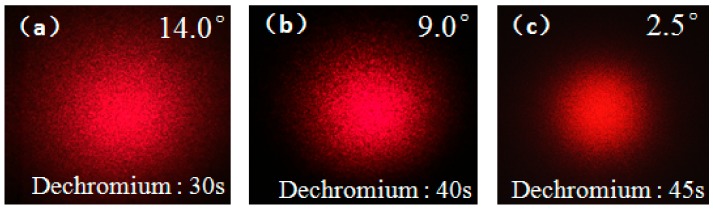
The scattering speckle patterns produced by the structures with different dechromium times: (**a**) 30 s; (**b**) 40 s; (**c**) 45 s.

The influence of different processing parameters on speckle divergence angle was analyzed. The same mask was used to prepare the same resist pattern structures. Then, the structures were immersed in dichromium for 30 s, 40 s, and 45 s to obtain chrome layer patterns with different thicknesses. Through the acidic etching, the final structures were achieved and the corresponding speckle divergence angles were analyzed as 14.0°, 9.0°, and 2.5°, respectively as shown in Figure 11. It was found that, with the increase of chromium corrosion time, the divergence angle of the final speckle decreases. This is because the different thicknesses of the chromium layers are etched in the process. The etched chromium layer is not dense, and hydrogen fluoride (HF) can penetrate through the gap and start etching the glass. The residual thickness of the etched chromium layer is also used as a masking layer for glass etching to slow down the stripping velocity of polygonal chromium masking layer during glass etching. If the chromium layer is thinner, the stripping velocity of the polygon chromium will be faster. The depth of HF etching glass will be shallower and the divergence angle becomes smaller. However, the chrome layer of the polygons cannot be stripped too fast, which will cause the final corrosion of the random microwells cannot be tightly connected, thus the final speckle will have zero-order light phenomenon. In quantum imaging, the existence of zero-order light will seriously affect the imaging quality.

Through adjusting the size of the polygons and gaps in the designed mask and corresponding process parameters to effectively control the size of the diameters and depths, the controllable divergence angle of the pseudo-thermal light source can be finally obtained. The component can replace the rotary ground glass in the traditional pseudo-thermal light sources to obtain the controllable divergence angle of the pseudo-thermal light.

## 4. Conclusions

In this paper, microwell arrays with randomly varying diameters have been successfully prepared to provide a new structural element for pseudo-thermal speckle light source. The microwell array with diameters randomly varying from 5 μm to 40 μm, height varying from 200 nm to 20 μm, was fabricated by photolithography combined with acid etching, which can be used for the generation of a pseudo-thermal light source in a controllable divergent angle.
